# A Direct Brain-to-Brain Interface in Humans

**DOI:** 10.1371/journal.pone.0111332

**Published:** 2014-11-05

**Authors:** Rajesh P. N. Rao, Andrea Stocco, Matthew Bryan, Devapratim Sarma, Tiffany M. Youngquist, Joseph Wu, Chantel S. Prat

**Affiliations:** 1 Department of Computer Science & Engineering, University of Washington, Seattle, Washington, United States of America; 2 Department of Psychology and Institute for Learning & Brain Sciences, University of Washington, Seattle, Washington, United States of America; 3 Department of Bioengineering, University of Washington, Seattle, Washington, United States of America; University of California, Irvine, United States of America

## Abstract

We describe the first direct brain-to-brain interface in humans and present results from experiments involving six different subjects. Our non-invasive interface, demonstrated originally in August 2013, combines electroencephalography (EEG) for recording brain signals with transcranial magnetic stimulation (TMS) for delivering information to the brain. We illustrate our method using a visuomotor task in which two humans must cooperate through direct brain-to-brain communication to achieve a desired goal in a computer game. The brain-to-brain interface detects motor imagery in EEG signals recorded from one subject (the “sender”) and transmits this information over the internet to the motor cortex region of a second subject (the “receiver”). This allows the sender to cause a desired motor response in the receiver (a press on a touchpad) via TMS. We quantify the performance of the brain-to-brain interface in terms of the amount of information transmitted as well as the accuracies attained in (1) decoding the sender’s signals, (2) generating a motor response from the receiver upon stimulation, and (3) achieving the overall goal in the cooperative visuomotor task. Our results provide evidence for a rudimentary form of direct information transmission from one human brain to another using non-invasive means.

## Introduction

Many of the greatest contemporary technological developments have centered on advancing human communication. From the telegraph to the Internet, the primary utility of these game-changing innovations has been to increase the range of audiences that an individual can reach.

However, most current methods for communicating are still limited by the words and symbols available to the sender and understood by the receiver. Even when they include non-verbal content (as in the case of visual and auditory information), communication constraints can be severe. A great deal of the information that is available to our brain is not introspectively available to our consciousness, and thus cannot be voluntarily put in linguistic form. For instance, knowledge about one’s own fine motor control is completely opaque to the subject [1], and thus cannot be verbalized. As a consequence, a trained surgeon or a skilled violinist cannot simply “tell” a novice how to exactly position and move the fingers during the execution of critical hand movements. But even knowledge that is introspectively available can be difficult to verbalize. Brilliant teachers may struggle to express abstract scientific concepts in language [2], and everyone is familiar with the difficulty of putting one’s own feelings into words. Even when knowledge can be expressed in words, one might face the hurdle of translating between the many existing spoken human languages. Can information that is available in the brain be transferred directly in the form of the neural code, bypassing language altogether? We explore this idea in the rest of this article.

The idea of direct brain-to-brain communication could potentially be achieved using a Brain-to-Brain Interface (BBI) [3]–[5]. A BBI rests on two pillars: the capacity to read (or “decode”) useful information from neural activity and the capacity to write (or “encode”) digital information back into neural activity. In recent years, we have witnessed incredible progress in these two capabilities with the development of Brain-Computer Interfaces, or BCIs [6], [7]. BCI researchers have demonstrated the possibility of decoding motor [8], visual [9] and even conceptual information [10] from neural activity via a range of recording techniques such as implanted electrodes [8], electrocorticography (ECoG, e.g., [11]), electroencephalography (EEG, e.g., [12]), functional MRI (e.g., [13]), and magnetoencephalography (MEG, e.g., [14]). A variety of stimulation techniques also exist that permit users to encode digital information into neural activity using implanted electrodes [15], [16], transcranial magnetic stimulation, (TMS, [17]) and focused ultrasound (FUS, [18]). Prominent examples of BCIs that use stimulation include the cochlear implant [15] and deep brain stimulators [16].

Given these advances in BCIs, two recent efforts have addressed the question of whether direct brain-to-brain communication is possible with the technology we have today. Pais-Vieira and colleagues [3] explored the possibility of directly connecting the brains of two awake and behaving rats. In their experiment, cortical microelectrode arrays recorded the neural activity of “encoder” rats performing either a motor task or a tactile stimulation task, and guided the stimulation of motor and sensory areas in the brains of “decoder” rats. Because the actions of “decoder” rats mimicked those of the original “encoder” rats, the authors concluded that information had to have been transferred between their brains. An alternative BBI was proposed by Yoo and colleagues [5], who successfully demonstrated the transmission of information from a human brain to a rat brain. In this case, visual evoked potentials in the human brain were recorded with EEG and translated into FUS-based stimulation of the part of motor cortex that controlled the tail of the anesthetized rat.

Both of these BBIs rely on stimulation technologies that are either invasive or experimental in humans, and thus are currently confined to animal models. In this paper, we report results from the first non-invasive BBI that can be safely applied to humans. Specifically, we show that it is possible to use EEG to decode motor intentions from a “sender” brain, and TMS to deliver an equivalent motor command to the motor cortex of a “receiver” brain, allowing the receiver to perform the hand movement that was intended by the sender. To test the feasibility and applicability of this procedure, a task was designed that required cooperative information sharing between pairs of participants along the BBI. The rest of the article describes the BBI in detail and presents in-depth results from 6 human participants who played the role of either sender or receiver of information in the BBI. Results from the first demonstration of this BBI were announced in an online report in August 2013 [19].

## Materials and Methods

### Human Subjects and Ethics Statement

Six participants (aged 21–38; see Table 1) took part in the experiment over the course of three months. All participants were recruited through word of mouth, were fully informed about the experimental procedure and its potential risks and benefits, and gave written consent prior to the beginning of the experiment. They were divided into three pairs, with one participant playing the role of the “sender” and one playing the role of the “receiver.” Because the TMS procedure is inherently more risky than the EEG procedure, participants were allowed to decide which role they wanted to play. To maintain their decision free of any external influence, all participants received monetary compensation that was independent of their role and proportional to the total amount of time devoted to the study.

**Table 1:** Participant demographics.

Pair	Role	Age	Gender
*Pair 1*	Sender	21	M
	Receiver	30	M
*Pair 2*	Sender	23	M
	Receiver	27	M
*Pair 3*	Sender	27	M
	Receiver	38	M

Both the experiment and its recruitment procedure were reviewed and approved by the Institutional Review Board of the University of Washington. The individuals in this manuscript have given written informed consent (as outlined in the PLOS consent form) to publish these case details.

### Experimental Task

**Figure 1:**
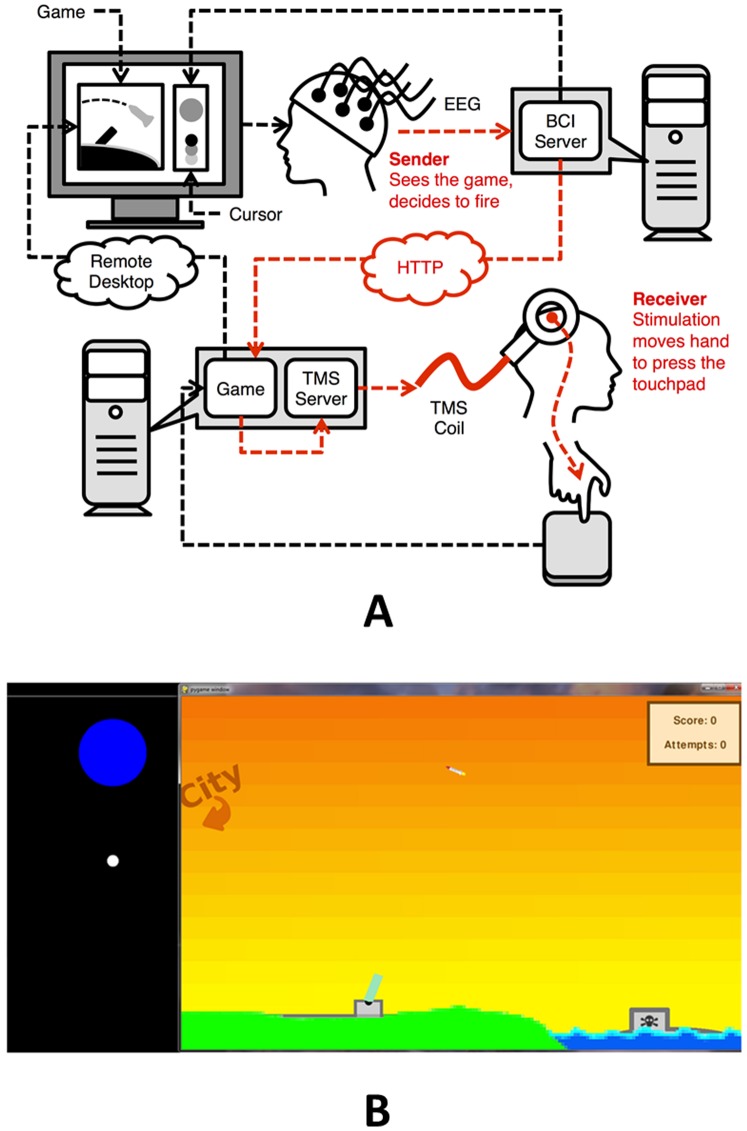
Experimental Set-Up. (A) Schematic diagram of set-up. Brain signals from one participant (the “Sender”) were recorded using EEG. When imagined hand movements were detected by the computer, a “Fire” command was transmitted over the internet to the TMS machine, which caused an upward movement of the right hand of a second participant (the “Receiver”), resulting in a press by the hand on a touchpad. This press triggered the firing of the cannon in the game seen by the Sender. Red lines mark the part of the architecture that corresponds to the direct brain-to-brain interface. (B) Screen shot from the game. In 50% of the trials, the pirate ship on the right side (skull-and-bones) shoots a rocket (top center) towards a city on the left. The Sender engages in motor imagery to move the white cursor on the left to hit the blue circular target in order to fire the cannon (bottom center) and destroy the rocket before it reaches the city. In the other 50% of the trials, a supply airplane moves from the right to the left side of the screen (not shown). The Sender rests in this case and refrains from imagery in order to avoid hitting the target.

During each experimental session, two participants had to carry out a specific task in the form of a series of consecutive trials of a computer game. The game was designed so that the two participants had to play cooperatively, and the required cooperation could only be achieved through direct brain-to-brain communication (Figure 1A). The goal of the game (Figure 1B) was to defend a city (located beyond the left visible part of the screen) from enemy rockets fired by a pirate ship on the lower right portion of the screen (represented by a skull-and-bones insignia). The rockets followed an arc trajectory, traversing the screen from the lower right to the upper left corner of the screen. A cannon, located in the lower center portion of the screen, tracked the rocket as it crossed the screen. To defend the city, the subjects had to fire the cannon by pressing a touchpad. If the cannon was fired before the moving rocket reached the city, the rocket was destroyed and the city was saved. In 50% of the trials, a friendly “supply airplane” flew across the screen instead of a pirate rocket. In such trials, participants had to avoid firing the cannon to let the supply airplane enter the city. Note that this task stresses the real-time nature of our BBI because the participants have to destroy the rocket *before* it crosses the screen for the trial to be successful.

### Brain-to-Brain Collaboration Between the Two Participants

**Figure 2:**
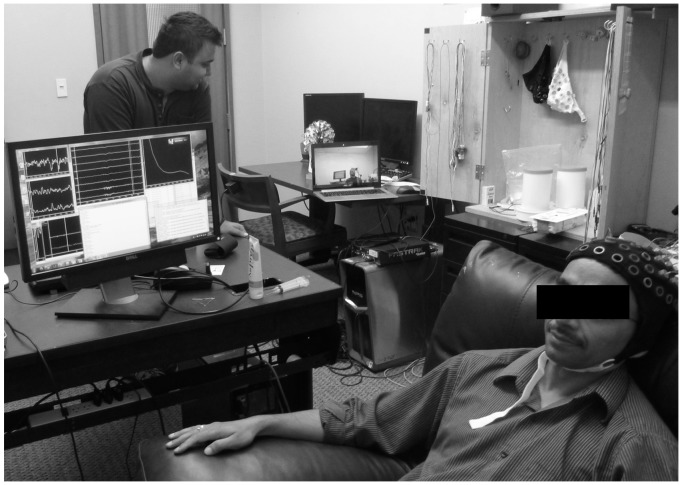
EEG Set-Up. EEG signals being recorded from a subject (the “Sender”) as the subject watches the computer game (the game screen is to the left and not shown in the picture). The larger screen displays EEG signals processed by the BCI2000 software. The smaller laptop screen placed further away is from the live Skype session and shows a “Receiver” subject in the TMS lab across the University of Washington campus. (Image from the pilot study referred to in the text).

The two participants were given different and complementary roles. One participant (henceforth, the “Sender”) was able to see the game on a computer screen, but was not provided with any input device to control the cannon (Figure 2, Sender watching the game screen, which is not shown). The second participant (henceforth, the “Receiver”) could use his/her right hand to press a touchpad, but could not see the game. The two participants were located in separate buildings on the University of Washington’s campus. Specifically, the Sender side was stationed in the Computer Science & Engineering building while the Receiver side was stationed in the Psychology building. The two buildings were located approximately 1 mile apart. The two participants could only communicate with each other through a brain-to-brain communication channel.

The brain-to-brain communication channel was built using two existing technologies: EEG for non-invasively recording brain signals from the scalp and TMS for non-invasively stimulating the brain (Figure 1A). During rocket trials, the sender conveyed the intent to fire the cannon by engaging in right hand motor imagery. Electrical brain activity from the Sender was recorded using EEG, and the resultant signal was used to control the vertical movement of a cursor (Figure 1B) – this allowed the subject to get continuous feedback about imagery performance. When the cursor hit the “Fire” target (a large blue circle) located at the top of the screen, the Sender’s computer transmitted a signal over the Internet to the Receiver’s computer. The two computers communicated using the standard hypertext transfer protocol (HTTP).

The Receiver’s computer was connected through a custom-made serial cable to a TMS machine. Whenever the Receiver’s computer received a fire command, a TMS pulse was delivered to a pre-selected region of the Receiver's brain. The stimulation caused a quick upward jerk of the Receiver's right hand, which was positioned above the touchpad. This up-down movement of the hand typically resulted in enough force to trigger a “click” event on the touchpad, causing the cannon in the computer game to be fired as requested by the Sender. In successful “supply airplane” trials, the Sender would rest and refrain from motor imagery, allowing the cursor to drift towards the bottom of the screen; in such trials, no signal was sent to the Receiver’s computer.

An analysis of the log times revealed that the transmission of information across the internet took approximately 10 ms, the activation of the TMS machine occurred approximately 1.4 ms later, the generation of the electromagnetic pulse occurred approximately 4.0 ms later, and the receiver’s entire motor response (corresponding the end of the downward trajectory of the hand, following the upward movement generated by the TMS) occurred on average 627.1 ms afterwards. Thus, the transmission of the signal along the BBI channel took ∼650 ms.

### Procedure

Each experiment consisted of two experimental blocks and two control blocks (see below), the order of which was randomized prior to the beginning of the experiment. The length of each block was initially set to 10 trials for Pair 1 to make sure that the experimental session could be completed within 1 hour, so as to minimize the discomfort for the TMS participant. Since the setup procedure was significantly faster than what we had initially estimated (based on our 2013 pilot study), the length of each block was extended to 16 trials for Pairs 2 and 3. Participants were told in advance of the presence of two conditions, but were not told to which condition each block belonged. Trials were separated by 2 seconds of set-up time plus a 20 second visual countdown. This large pause prevented two consecutive TMS pulses from being delivered less than 20 seconds apart, thus reducing the maximum amount of magnetic stimulation delivered to the Receiver to a level well below the strictest safety guidelines [20], and setting the upper limit of information throughput to 0.05 bits per second.

During experimental blocks, the non-invasive brain-to-brain channel was fully operational. However, during control blocks the brain-to-brain channel was made non-operational by changing the coil position so that the TMS pulse could not cause the desired movement of the right hand. Note that the electromagnetic pulse was still delivered at the same intensity, thus making the two conditions identical but for the target location. As will be clear in the discussion of the results, the control condition affects the performance of the receiver but does not have detectable effects on the sender’s behavior.

### EEG Procedure

Participants playing the role of the Sender came in for two consecutive sessions: a training session and the BBI experimental session. During both sessions, electrical signals were recorded at a frequency of 512 Hz from the Sender's scalp via a 64-channel Ag/AgCl electrode cap (actiCAP, Brain Products GmBH, Gilching, Germany) and amplified using gUSBamps (Guger Technologies, Austria). A Laplacian spatial filter [7] was used to reduce artifacts common to nearby electrodes and emphasize local activity. Signal processing and data storage were managed through the BCI2000 software package.

**Figure 3:**
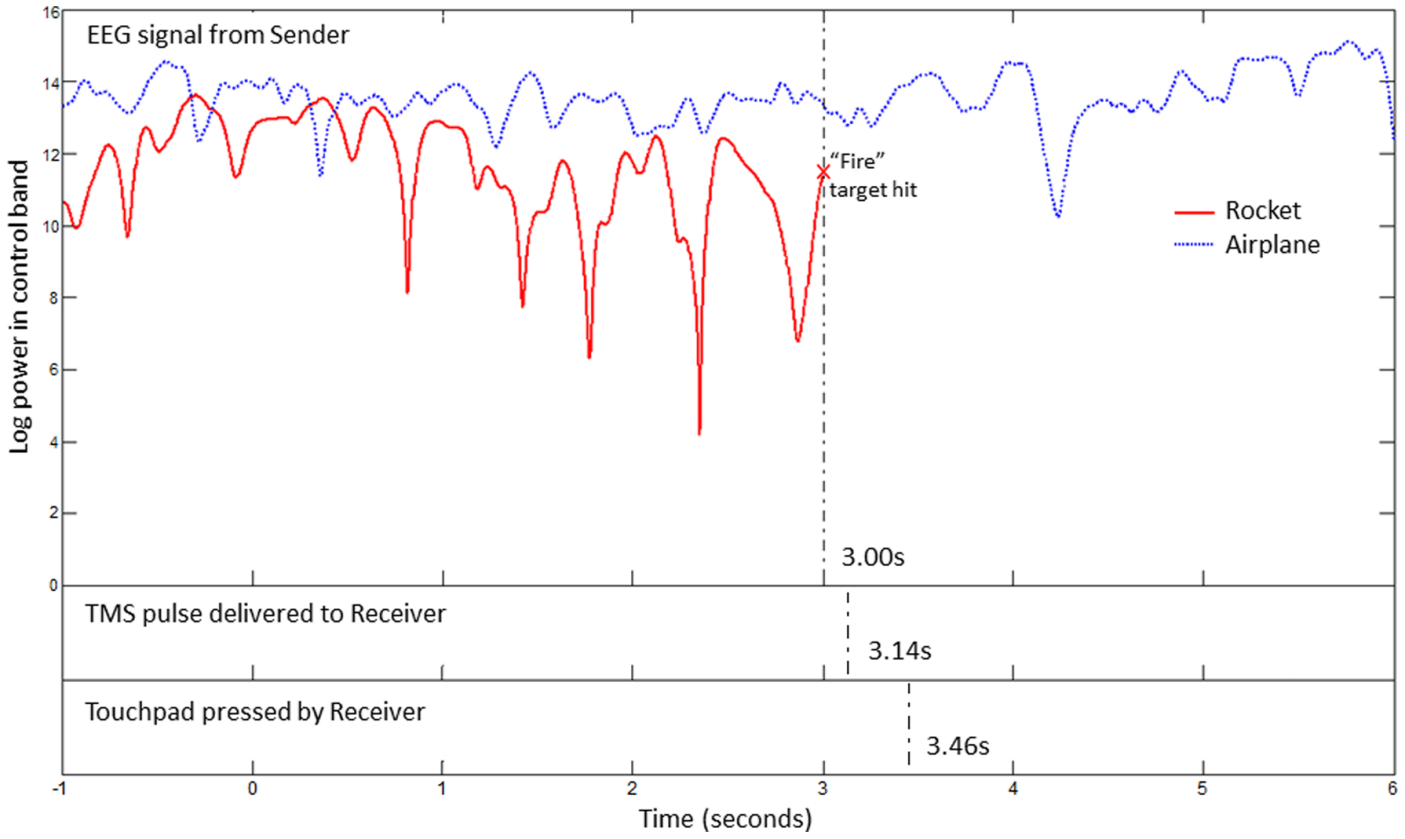
EEG Traces during the Two Trial Types and Timing of Information Transfer from Sender to Receiver during a Rocket Trial. EEG signal during one rocket trial (red trace) and one airplane trial (blue trace) from the Sender in Pair 1 is shown. The traces demonstrate suppression of power in the mu control band (11–13 Hz) during the rocket trial due to motor imagery. Dashed vertical lines mark timestamps of key events in the transfer of information in the BBI from Sender to Receiver during the rocket trial.

Changes in the “mu” band (typically 8–12 Hz) have long been linked to motor imagery signals and used in BCIs (for an introduction, see [6], [7]). During the training session, subjects learned to control the vertical movement of a 1-D cursor by imagining right hand movement. The power in a low frequency band (the “mu” band) was computed across the electrodes and the electrode most correlated with the subject's motor imagery during an initial training period was selected as the control electrode for the task. The computer translated the power in the mu band to vertical movement of a cursor, which provided visual feedback to the sender. Specifically, the decrease in power that accompanied right hand motor imagery was mapped to upward movement of the cursor, while a lack of suppression in the mu band caused downward cursor movement (see Figure 3 for an example). All the participants underwent the same amount of training. Although participants differ in their response to training, and it is reasonable to assume that increased training may improve a Sender’s subsequent performance, we did not make any attempt to optimize or manipulate the duration of the training sessions.

During the BBI experimental session, a monitor displayed both the cursor window and the cannon game (Figure 1B). Depending on the type of projectile in each trial, the Sender modulated activity in the mu band to guide the cursor to either the “Fire” target at the top of the screen or towards the bottom of the screen.

Note that this procedure tracks the Sender’s *intention* of moving the right hand and not the hand movement itself, which was, in fact, not necessary to trigger the remote action. Motor imagery is frequently adopted in BCIs developed for disabled patients, who have retained control of such imagery but whose motor activity is disrupted [6], [7]. Imagery is central to the demonstration of brain-to-brain communication, as the movement intention that is initially imagined in the Sender’s brain is remotely executed by the Receiver’s brain.

### TMS Procedure

Participants playing the role of the Receiver came in for two consecutive sessions. During the first session, as part of informed consent, they were asked to complete a TMS safety screening questionnaire, aimed at identifying potential conditions (such as family history of seizures or frequent migraines) that might represent potential risk factors for adverse side effects of TMS. Only one potential candidate was rejected for failing the safety questionnaire (due to frequent migraines).

Participants who passed the safety questionnaire underwent a TMS parameter estimation session, whereby the appropriate stimulation site and intensity was identified. The procedure worked as follows. The participant was asked to wear a tight-fitting swim cap, where the location of the inion and the vertex were identified using the 10–20 system procedure [7]. A 4×4 grid of dots were marked on the upper left region of the vertex, each dot placed at a distance of 1 cm from its neighbors. Each dot location was then stimulated in sequence, using a single pulse delivered by a 90 mm MagStim circle coil connected to a Super Rapid^2^ magnetic stimulator (MagStim, UK). This search procedure continued until an ideal position was found to stimulate the motor region that controls the *extensor carpi radialis*. Notice that, because this muscle *extends* the wrist, it produces an *upward* movement of the hand.

**Figure 4:**
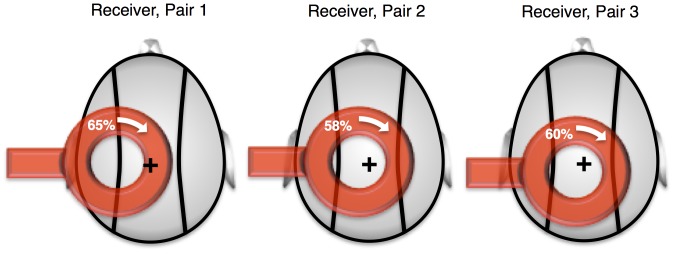
Stimulation Parameters for the Three Receivers. The figure represents the approximate position of the TMS circle coil (in red) on the head of the three participants. The “+” sign represents the location of the vertex. The white arrow shows the direction of the inducing current in the coil; the numbers represent the intensity of the magnetic stimulation used for each receiver. As is commonplace in the TMS literature, the intensity of the stimulation is expressed as a percentage of the maximum stimulator output (which was 2.0 T).

The circle coil was always placed so that it was flush against the head, the current was flowing in clock-wise direction (“B” side), and the coil handle pointed horizontally and leftwards from the participant’s head (Figure 4). The stimulation intensity was estimated as the minimum amount of power that was needed to solicit a consistent upward response of the hand. Once identified, the coil position and the stimulation intensity were marked on the cap, and the cap was put in a sealed envelope to be re-used in the experimental session. The parameter estimation session lasted between 10 and 30 minutes. The stimulation parameters for the three participants who played the role of receivers are given in Figure 4. The intensity of the stimulation for each subject is expressed as a percentage of the maximum stimulator output. The maximum intensity of the electric field for our TMS equipment is 530 V/m, and with our coil, the maximum intensity of the induced magnetic field is 2.0 T.

**Figure 5:**
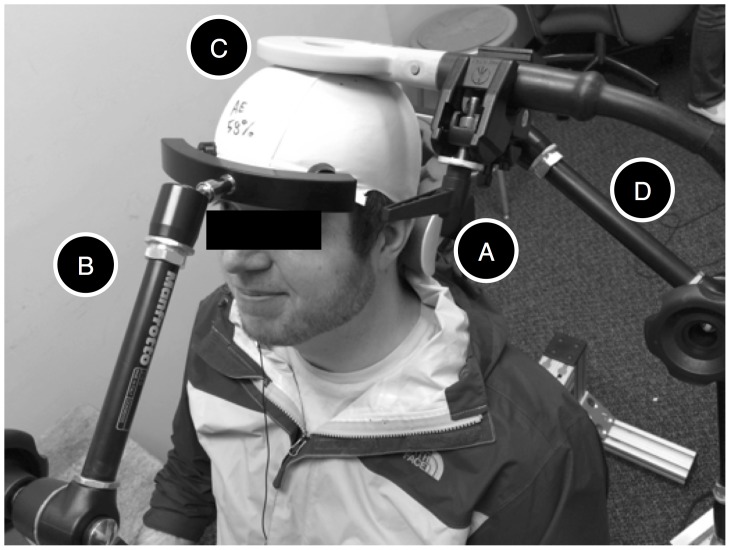
TMS Set-Up. During the experiment, the Receiver was accommodated on a BrainSight chair, with the back of the head resting against a neckrest (A) and kept in place by an adjustable arm with padded forehead prongs (B). A 90 mm circular TMS coil (C) was kept in place by an articulated arm (D). During the experiment, the receiver wore noise-cancellation earphones (not shown) while listening to a selection of music or to an audiobook of his/her own choice.

During the experimental session, the returning participant wore the same cap while sitting on an anatomical chair designed for TMS (BrainSight, Rogue Resolutions, Montreal, CA). The participant’s head was accommodated on a neck rest (Figure 5A) and kept in position by an adjustable arm equipped with padded forehead prongs (Figure 5B). The participant’s left arm was accommodated on the chair’s armrest. The chair’s right armrest was removed, and the participants’ right arm was placed on an adjustable table, so that the four non-opposable fingers of the right hand rested on a wireless touchpad (Logitech T650, Morges, Switzerland) connected to the Receiver’s computer. The Sender’s intention to move the hand caused the TMS machine to fire a single pulse at the intensity that was established during the parameter estimation session, Note that because the TMS pulse causes an upward jerk of the hand, the touchpad is actually pressed during the downward part of the moment, when the hand falls back in position. Before the experiment, the TMS coil (Figure 5C) was mounted on a dual rod articulated arm (Manfrotto, Cassola, Italy: Figure 5D) connected to the left swinging arm of the anatomical chair.

To keep the Receiver subjects blind to the course of the experiment, the TMS room was set up so that they were facing a blank wall with their back to the experimenter and the experimental equipment, including the TMS computer and the TMS device. In addition, participants were required to wear a pair of noise cancellation earphones (Bose QuiteComfort 20/20i, Framingham, MA). Before the beginning of the experiment, participants were instructed to bring either music or an audiobook of their choice to be played through the earphones. As a result, the TMS participants were not given any visual or acoustic cues about the experiment. At the beginning of each new block, the TMS experimenter moved the TMS coil away from the participant’s head, and changed the position of the coil. During experimental blocks, the coil was placed in the exact same position determined during the parameter estimation session. During control blocks, the position of the coil was changed so that, with the same power output, it could not trigger a hand response. Specifically, the coil was simply rotated 180 degrees on the axis of its handle, so that its electrical current was flowing in counter-clockwise fashion (“A” side facing up). None of the participants could distinguish between the two coil positions.

## Results

### Overall Accuracy in the Experimental Task

Because our task was designed so that it could be carried out successfully only if participants cooperated though the BBI, one way to gauge the efficacy of our BBI was to examine each pair’s accuracy during the game, and specifically, to compare the pair’s accuracy during the experimental and the control blocks. Overall, the three pairs of subjects correctly identified and destroyed 83.3%, 25.0%, and 37.5% of the rockets respectively during the experimental blocks, and 0% of the rockets during the control blocks (see Table 2). The difference between the two conditions indicates that the BBI was crucial in enabling the two participants to collaborate. Figure 3 shows examples of two successful BBI trials by Pair 1.

**Table 2:** Percentage of rockets and planes hit by pair.

	Condition	Pair 1	Pair 2	Pair 3
*% Rockets Hit*	Experimental	83.33%	25.00%	37.50%
	Control	0.00%	0.00%	0.00%
*% Planes Hit*	Experimental	37.50	18.75%	0.00%
	Control	0.00%	0.00%	0.00%

**Figure 6:**
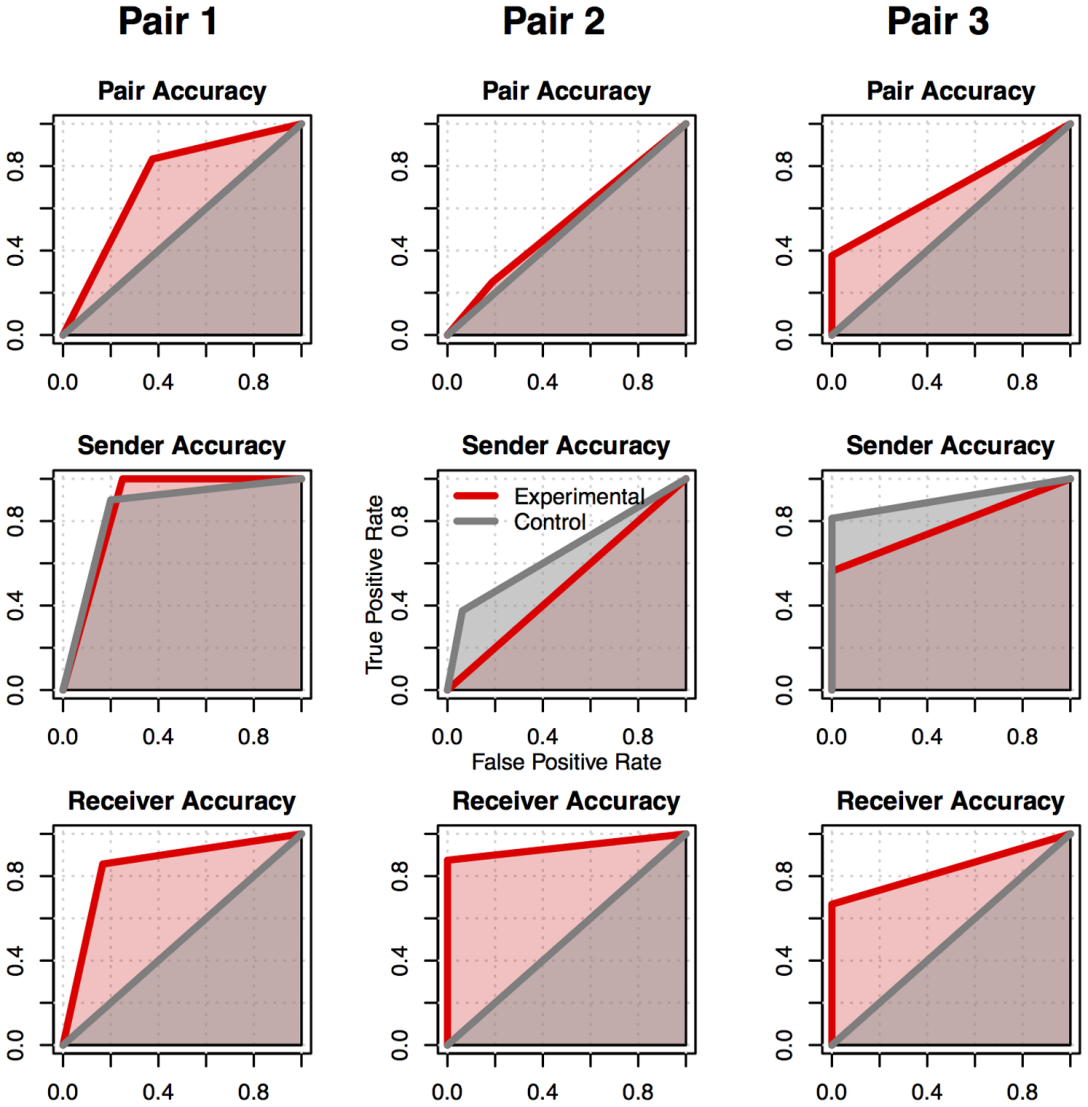
BBI Accuracy. ROC curves for each of the three pairs of subjects (columns), presented in terms of overall pair accuracy in the game (top panels), accuracy of the Sender (middle panels), and accuracy of the Receiver (bottom panels). Red lines and areas represent the experimental conditions, while grey lines and areas represent the control conditions.

The percentage of rockets that were identified and destroyed provides only a partial measure of the participants’ behavior. Each experimental trial can be categorized as a “true positive” (a rocket correctly destroyed), a “false positive” (supply airplane that was mistakenly destroyed), a “false negative” (a rocket that was not destroyed), or a “true negative rejection” (supply airplane that was allowed to fly by). Therefore, the collaboration between the two subjects in the three pairs can be quantified using signal detection theory, and in particular, by calculating the area under the corresponding Receiver Operating Characteristic (ROC) curve [21]. Because the ROC curve plots the proportion of true positives against the proportion of false positives, optimal performance corresponds to an area of 1, and chance performance corresponds to an area of 0.5. The results of ROC analysis for the BBI interface are visually represented in the first row of Figure 6. In each plot, the red line represents the ROC curve for the experimental condition, and the grey line represents the ROC curve for the control condition. From the ROC curves, it is apparent that Pairs 1 and 3 were overall successful in cooperatively playing the game (i.e., the area under the red ROC curve was larger than 0.5, and larger than the area under the grey ROC curve), while Pair 2 was not (the grey and red curves have approximately the same areas), due to poor discriminability of the Sender’s EEG signals as discussed below.

### Behavioral and EEG Data for the Senders

Because of the cooperative nature of the task, the overall performance of the pair is constrained by the performance of both the sender and the receiver. Thus, poor performance of the pair might not accurately reflect the efficacy of the BBI *per se*, but only of one of the two participants. For this reason, we estimated the ROC curves for each sender and receiver separately. In the case of the sender, the ground truth is represented by the object on the screen (whether it is a supply plane or rocket) and the response by the participant’s success in moving the vertical cursor to the target. Thus, moving the cursor upward in response to a rocket represents a true positive, while moving the cursor upward in response to a plane a false positive. In the case of the receiver, the ground truth is the TMS pulse, and the desired motor response is the corresponding touchpad press. Thus, a touchpad press in response to a TMS pulse is a true positive whereas a press without a TMS pulse is a false positive.

**Figure 7:**
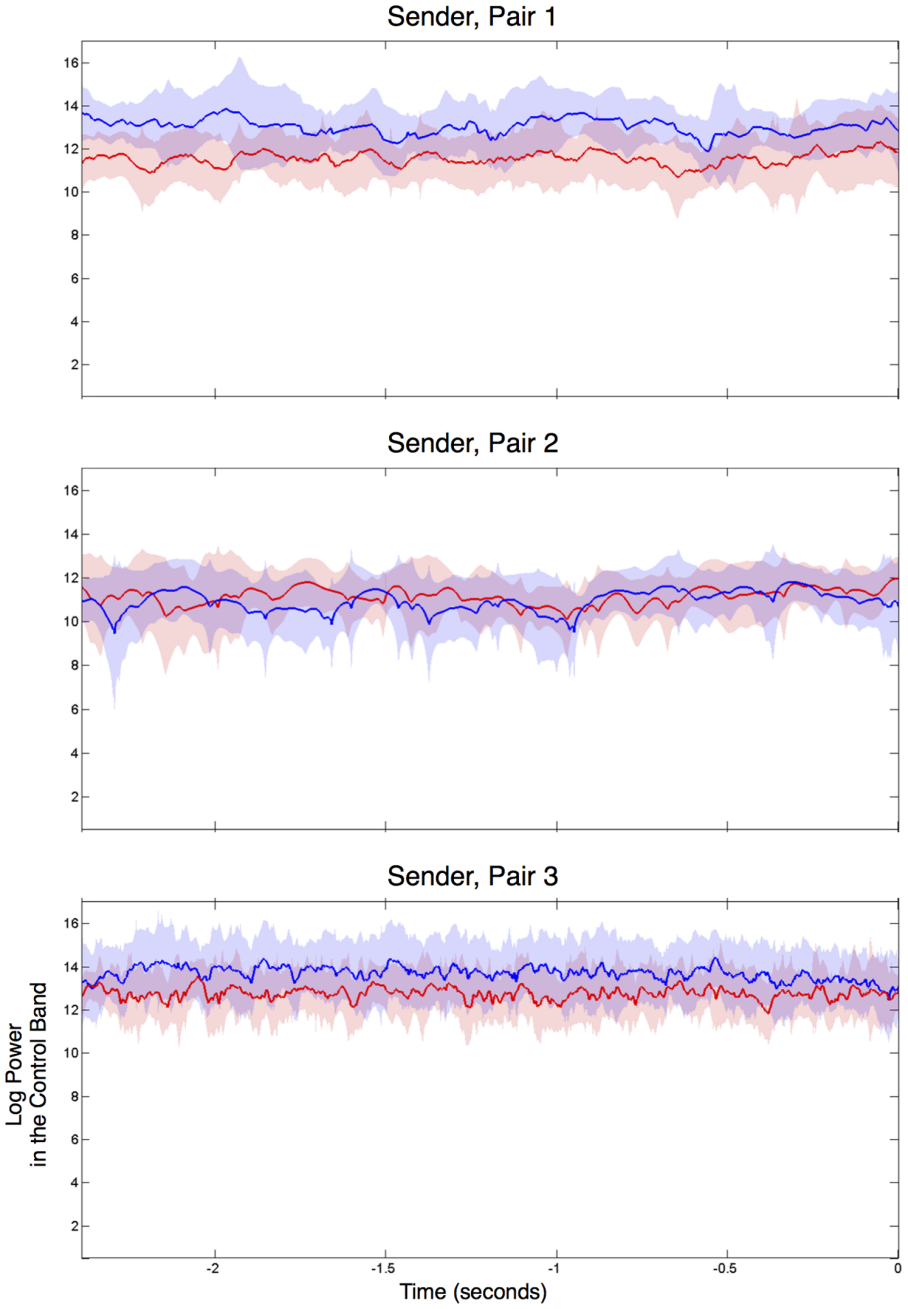
Task-related EEG Activity for the Senders in the Three BBI Pairs. Each panel shows the log power (mean +/−1 standard deviation) in the control band for a Sender during the final 2.5s before the cursor hit the target for all rocket trials (red). For comparison, data from airplane trials for the same time period are shown in blue. The control bands were as follows: Pair 1∶11–13 Hz; Pair 2∶18–20 Hz; Pair 3∶11–28 Hz. There is a clear separation in EEG control signals for the two types of trials for the Senders in Pairs 1 and 3, but not in Pair 2.

When the ROC curves for the Sender and the Receiver are plotted separately (Figure 6, middle and bottom rows), it becomes apparent that Pair 2′s poor performance was due to the Sender’s failure to consistently move the cursor upward to the “Fire” target during rocket trials. This is confirmed by an inspection of the task-related EEG activity of the three senders, as summarized in Figure 7. Each panel in the figure plots the average power of a Sender’s brain activity in the subject-specific mu control band during the 2.5 seconds before the cursor hit the target and a “Fire” command was sent to the TMS machine. In the figure, data from Rocket trials are plotted in red while data from Airplane trials (during the same time period) are plotted in blue. Notice how, in the 2.5 seconds preceding a response, the EEG activity for rocket versus airplane trials are well separated for the Senders in Pair 1 (top panel) and Pair 3 (bottom panel). This separation is indicative of the Sender’s capacity to control the cursor’s position by engaging in motor imagery and successfully reducing mu band activity during the rocket trials. In contrast, EEG activity was essentially identical for rocket versus airplane trials in the case of Pair 2 (middle panel), suggesting that the Sender, though successful in completing the initial training session, was unable to properly control the cursor during the BBI experiment, resulting in poor overall BBI performance for Pair 2.

### Behavioral Data for the Receivers

The performance of the three receivers was close to optimal in all the experimental blocks (Figure 6, third row). Thus, even when a Sender’s EEG signals could not successfully discriminate between planes and rockets (as in the case of Pair 2), the BBI system worked properly, and the two subjects could still collaborate on the task (albeit with low accuracy).

In contrast to the senders, the receivers’ performance was at a minimum during the control condition (corresponding to 0% of rockets correctly identified: see Table 2), reflecting the expected lack of response when the brain-to-brain channel was blocked. Notice that while the receivers’ performance varied as a function of the experimental condition (as evidenced by the difference between red and grey curves in the bottom row of Figure 6), the performance of the senders was unaffected by this manipulation, reflecting the fact that the manipulation of the BBI condition did not affect their behavior. This fact rules out the possibility that the apparent drop in performance during the control condition was due to the Sender not responding.

Finally, it is worth noting the extremely low rates of false positives across the receivers’ responses (only one response was recorded in the absence of TMS stimulation), as well as the complete lack of touchpad presses during the control blocks. These facts lend support to our statement that the Receiver could receive information only through the BBI. If the Receiver was responding based on an arbitrary strategy, or responding to some environmental cue (e.g., residual noise of the TMS pulse), then at least *some* false positives would be expected, at least at the beginning of the control blocks.

### Measures of BBI Efficacy

A more specific measure of the efficacy of the BBI is the degree to which a behavioral response of the Receiver can be ascribed to a pulse that was sent by the Sender, rather than to chance or response guessing. This measure can be computed by creating two binary vectors *S* and *R* for each block, each element of which corresponds to a single trial. Of these two vectors, *S* represents the Sender’s performance, and contains 1 s if the Sender delivered a “Fire” command to the Receiver, and 0 s if no “Fire” command was sent. The Receiver’s vector *R*, on the other hand, contains 1 s if the Receiver pressed the touchpad during the trial, and 0 s if the touchpad was not pressed. The efficacy of the BBI is proportional to the extent to which *R* is explained by *S*, which in turn can be measured by the slope coefficient β in a linear regression model *R* = α+β*S. Note that, if the Receiver responds randomly, then the two vectors are completely uncorrelated, and β = 0. On the other hand, when the two vectors are identical and perfectly correlated, then β = 1.

**Figure 8:**
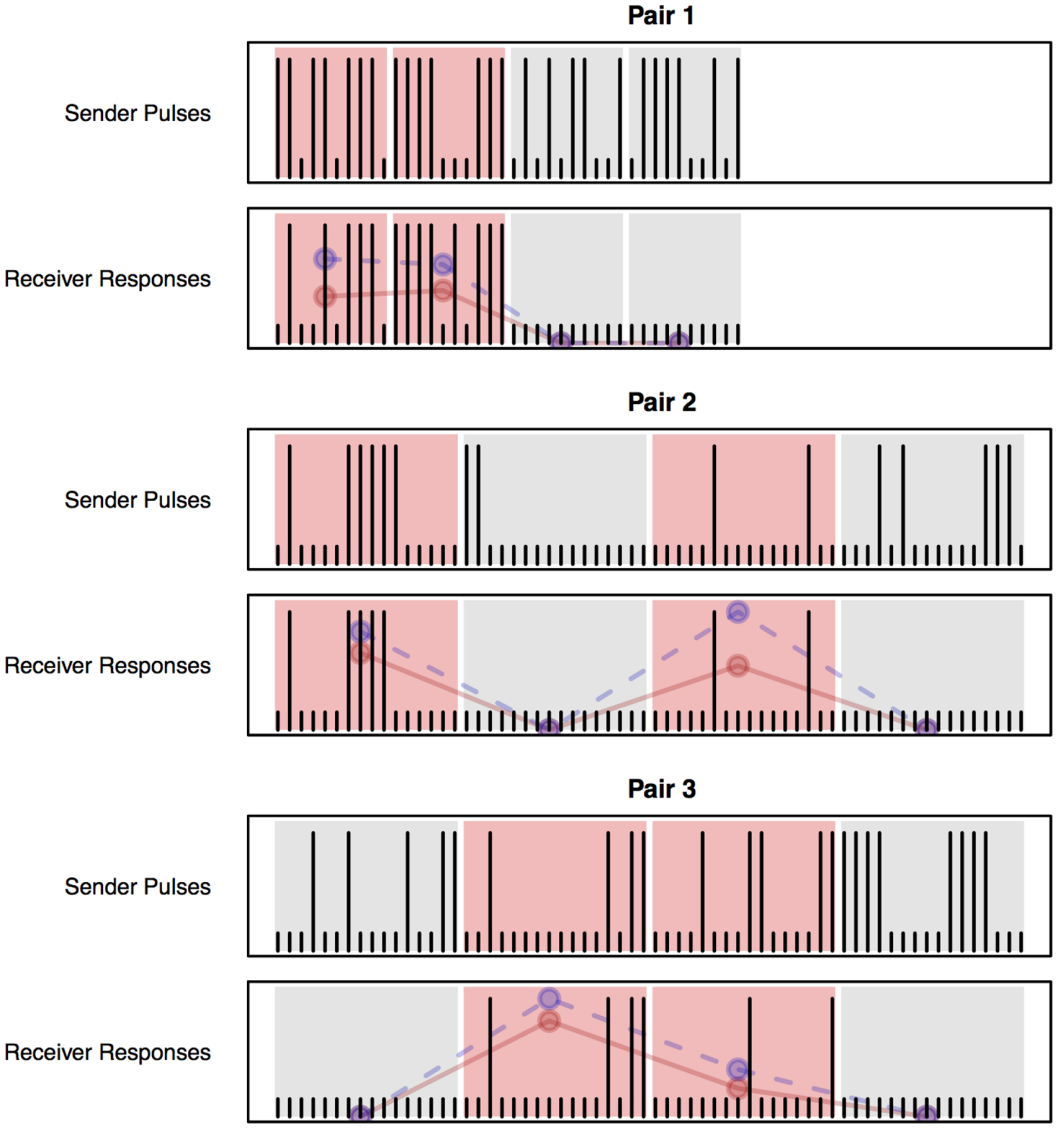
Response Vectors for the Sender and Receiver across Three Pairs . Each vertical tick represents a trial; long lines represent behavioral responses. Experimental blocks are marked by a red background; control blocks by a grey background. The blue dashed line represents the block-specific value of the regression coefficient β (see text for details); the red line represents the block-specific value of the mutual information between the two vectors. Note that in all six experimental blocks, the value of β was significantly greater than zero while in all control blocks, the value was zero. Likewise, about 4 to 13 bits of information (see Table 3) were transferred from one brain to another during experimental blocks, compared to zero bits in the control blocks.

Figure 8 visually depicts the *S* and *R* vectors across all pairs of participants and all blocks, as well as the values of β for each block (blue dashed line). In all six experimental blocks, the value of β was significantly greater than zero, ranging from β = 0.4 (Pair 3, second experimental block, *F*(1,14) = 6.42, *p* = 0.02) to β = 1 (Pair 3, first experimental block). Also, β = 0 in *all* six control blocks. Thus, by establishing that the effect of the Sender’s pulses on the Receivers’ responses is greater than expected by chance, we are also implicitly establishing that the effect is significantly greater in the experimental conditions than in the control conditions, as would be expected from a working BBI system.

Finally, it is useful to calculate the amount of information that was effectively transmitted between a Sender and a Receiver. This can be estimated by calculating the mutual information [22] between the Sender and Receiver response vectors, i.e. *I*(*S*, *R*) (see Figure 8, red line, and Table 3). The value of *I*(*S*, *R*) ranges between 0 and 1, and represents the mean number of bits per element in a sequence. Thus, the total amount of information transferred during a block can be estimated by multiplying *I*(*S*, *R*) by the number of trials within that block. The results of these calculations, given in Table 3, show that no information was transferred during the control blocks, while about 4 to 13 bits were transferred from one brain to the other during each experimental block.

**Table 3:** Mutual information between Sender and Receiver response vectors across the different conditions and pairs of participants.

	Experimental Blocks		Control Blocks	
	First	Second	First	Second
*Pair 1*	0.39 (3.9)	0.45 (4.5)	0.00 (0.00)	0.00 (0.00)
*Pair 2*	0.65 (10.4)	0.54 (8.64)	0.00 (0.00)	0.00 (0.00)
*Pair 3*	0.81 (12.96)	0.24 (3.84)	0.00 (0.00)	0.00 (0.00)

Values in parenthesis indicate the total number of bits transferred during the corresponding block.

## Discussion and Conclusion

Our results show that information extracted from one brain using EEG can be transmitted to another brain using TMS, ultimately allowing two humans to *cooperatively perform a task* using only a direct brain-to-brain interface (BBI) as a channel of communication. In conjunction with our pilot demonstration in 2013 [19], these results represent the first working BBI in humans.

We believe that our results are noteworthy on at least three fronts. First, they show that current technology is sufficient to develop devices for rudimentary brain-to-brain information transmission in humans. Such devices (which have been long cherished by science fiction writers) have the potential to not only revolutionize how humans communicate and collaborate, but also open a new avenue for investigating brain function. Second, our results show that working BBIs can be built out of non-invasive technologies. Because non-invasive technologies are currently simpler and safer for humans than invasive, surgically implanted devices, they have a potentially wider range of applicability, and could be used to develop BBIs in humans for a diverse array of tasks compared to animal BBIs. Third, by demonstrating a proof-of-concept BBI in humans, our results highlight the need for accelerating conversations between ethicists, neuroscientists, and regulatory agencies on the ethical, moral, and societal implications of BBIs whose future capabilities may go well beyond the rudimentary type of information transmission we have demonstrated here.

In comparison to invasive tools for stimulation [15], [16], non-invasive technologies, such as TMS or transcranial current stimulation (tCS: [23]) are currently more limited in both the number of available stimulation sites and the spatial resolution of stimulation targets. Thus, the development of BBIs that can truly replace or augment current means of human communication depends on significantly improving these technologies, or developing alternatives (such as focused ultrasound (FUS): [3], [18]). However, current established technologies like TMS or tCS could still be used in interesting ways beyond what we have demonstrated here. For example, rather than focusing on the transfer of motor intention information, TMS could theoretically be applied to almost any area of the cortex that is close to the skull, e.g., primary visual cortex [24]. Indeed, after this paper was submitted, researchers from a company called Starlab reported results from a BBI that builds on our idea of combining EEG and TMS but uses visual rather than motor stimulation. Their BBI, which was tested with only 1 sender, operates in an asynchronous mode using email: the sender does not receive any feedback from the receiver. The sender-receiver pair are therefore not cooperating in real-time to solve a task as in our case.

Another viable direction for future experiments is the possibility of using non-invasive technologies to implement one-to-many or many-to-one BBIs, whereby a signal from a single sender is broadcasted to multiple receivers, or multiple senders can submit signals to the same receiver [4]. Such a scenario is indeed already possible within our software architecture (Figure 1A).

Because the neural underpinnings of sensorimotor information are much better understood than those of conceptual and abstract information [25], [26], BBIs in the near future will likely be limited to transmitting visual, auditory, or motor information. In the long term, the development of more powerful BBIs will be predicated on understanding how abstract thoughts and complex cognitive information are encoded within distributed patterns of neural activity in the human brain. We see this as both a challenge and an opportunity for future research.
